# The PDZ-Binding Motif of Severe Acute Respiratory Syndrome Coronavirus Envelope Protein Is a Determinant of Viral Pathogenesis

**DOI:** 10.1371/journal.ppat.1004320

**Published:** 2014-08-14

**Authors:** Jose M. Jimenez-Guardeño, Jose L. Nieto-Torres, Marta L. DeDiego, Jose A. Regla-Nava, Raul Fernandez-Delgado, Carlos Castaño-Rodriguez, Luis Enjuanes

**Affiliations:** Department of Molecular and Cell Biology, Centro Nacional de Biotecnología (CNB-CSIC), Darwin 3, Campus Universidad Autónoma de Madrid, Madrid, Spain; Mount Sinai School of Medicine, United States of America

## Abstract

A recombinant severe acute respiratory syndrome coronavirus (SARS-CoV) lacking the envelope (E) protein is attenuated *in vivo*. Here we report that E protein PDZ-binding motif (PBM), a domain involved in protein-protein interactions, is a major determinant of virulence. Elimination of SARS-CoV E protein PBM by using reverse genetics caused a reduction in the deleterious exacerbation of the immune response triggered during infection with the parental virus and virus attenuation. Cellular protein syntenin was identified to bind the E protein PBM during SARS-CoV infection by using three complementary strategies, yeast two-hybrid, reciprocal coimmunoprecipitation and confocal microscopy assays. Syntenin redistributed from the nucleus to the cell cytoplasm during infection with viruses containing the E protein PBM, activating p38 MAPK and leading to the overexpression of inflammatory cytokines. Silencing of syntenin using siRNAs led to a decrease in p38 MAPK activation in SARS-CoV infected cells, further reinforcing their functional relationship. Active p38 MAPK was reduced in lungs of mice infected with SARS-CoVs lacking E protein PBM as compared with the parental virus, leading to a decreased expression of inflammatory cytokines and to virus attenuation. Interestingly, administration of a p38 MAPK inhibitor led to an increase in mice survival after infection with SARS-CoV, confirming the relevance of this pathway in SARS-CoV virulence. Therefore, the E protein PBM is a virulence domain that activates immunopathology most likely by using syntenin as a mediator of p38 MAPK induced inflammation.

## Author Summary

SARS-CoV caused a worldwide epidemic infecting 8000 people with a mortality of about 10%. A recombinant SARS-CoV lacking the E protein was attenuated *in vivo*. The E protein contains a PDZ-binding motif (PBM), a domain potentially involved in the interaction with more than 400 cellular proteins, which highlights its relevance in modulating host-cell behavior. To analyze the contributions of this motif to virulence, recombinant viruses with or without E protein PBM were generated. Recombinant SARS-CoVs lacking E protein PBM caused minimal lung damage and were attenuated, in contrast to viruses containing this motif, indicating that E protein PBM is a virulence determinant. E protein PBM induces the deleterious exacerbated immune response triggered during SARS-CoV infection, and interacts with the cellular protein syntenin, as demonstrated using proteomic analyses. Interestingly, syntenin redistributed from nucleus to cytoplasm during SARS-CoV infection, activating p38 MAPK and triggering the overexpression of inflammatory cytokines. Furthermore, silencing of syntenin using siRNAs led to a decrease in p38 MAPK activation. In addition, administration of a p38 MAPK inhibitor led to an increase in mice survival after SARS-CoV infection. These results indicate that syntenin and p38 MAPK are potential therapeutic targets to reduce the exacerbated immune response during SARS-CoV infection.

## Introduction

Severe acute respiratory syndrome coronavirus (SARS-CoV) was identified as the etiological agent of a respiratory disease that emerged in Southeast China at the end of 2002. SARS-CoV spread to more than 30 countries within six months, infecting 8000 people with an average mortality of 10% [1]. After July 2003, only a few community and laboratory-acquired cases have been reported (http://www.who.int/csr/sars/en/). Nevertheless, coronaviruses, including those similar to the strain that caused the epidemic, are widely disseminated in bats circulating all over the world, making a future outbreak possible [2]–[5]. In September 2012, a novel coronavirus, named Middle East respiratory syndrome coronavirus (MERS-CoV) was identified in two persons with severe respiratory disease [6], [7]. By now, 701 laboratory-confirmed MERS-CoV cases, including 249 deaths, have been diagnosed in several countries (http://www.who.int/csr/don/2014_06_16_mers/en/). Most patients reported respiratory disease symptoms, occasionally accompanied by acute renal failure [8]. A better understanding of the molecular mechanisms underlying the virulence of these highly pathogenic coronaviruses will facilitate the development of therapies to alleviate or prevent the impact of coronavirus infection on human health.

SARS-CoV belongs to the *Coronavirinae* subfamily, genus *β* and is an enveloped virus with a single-stranded positive sense 29.7 kb RNA genome [9]. SARS-CoV envelope (E) protein is a small integral membrane protein of 76 amino acids that contains a short hydrophilic amino-terminus followed by a hydrophobic region, and a hydrophilic carboxy-terminus [10]. The hydrophobic region forms at least one amphipathic α-helix that oligomerizes to form an ion-conductive pore in membranes [11]–[13]. E protein is present within virions in very small amounts, however it is abundant in the infected cells [14], and it is mainly localized in the endoplasmic reticulum Golgi intermediate compartment (ERGIC), where it actively participates in virus budding, morphogenesis and trafficking [15]–[17]. Interestingly, SARS-CoV lacking the E protein was attenuated in different animal models, such as hamsters, transgenic mice that expressed the SARS-CoV receptor, human angiotensin converting enzyme 2 (hACE-2), and conventional mice using a mouse adapted SARS-CoV [18]–[21], indicating that SARS-CoV E gene may be a virulence factor. We have previously shown that SARS-CoV E protein increased the apoptosis and reduced the stress response induced after SARS-CoV infection [22].

Transient expression of SARS-CoV E protein in trans showed that the protein associated with *Caenorhabditis elegans* lin-7 protein 1 (PALS1), a tight junction-associated protein, is an E protein interacting partner [23]. PALS1 bound E protein through the post-synaptic density protein-95/discs Large/zonula occludens-1 (PDZ) domain of PALS1 [23], which recognized the last four carboxy-terminal amino acids of E protein that form a type II PDZ-binding motif (PBM) with the consensus sequence X-φ-X-φ_COOH_ (where X represents any amino acid and φ is a hydrophobic residue, usually V, I or L) [24]. However, the relevance of this interaction during virus infection and its impact on virulence *in vivo* was not tested.

PDZ domains are protein–protein recognition sequences, consisting of 80–90 amino acids that bind to a specific peptide sequence (PBM), usually located at the end of the carboxy-terminus of a target protein [25]–[27]. Proteins containing PDZ domains are typically found in the cell cytoplasm or in association with the plasma membrane and play a role in a variety of cellular processes of significance to viruses, such as cell-cell junctions, cellular polarity, and signal transduction pathways [28]. PDZ domains are found in thousands of proteins and are widespread in eukaryotes and eubacteria [29]. Just in the human genome, there are more than 900 PDZ domains in at least 400 different proteins [30]. These protein-protein interactions modulate cellular pathways influencing viral replication, dissemination in the host or pathogenesis [28].

As previously described, SARS-CoV E protein contains a PBM [23]. However, the relevance of this motif in the context of infection and its role in virus pathogenesis has not been elucidated. In this study, we have identified the SARS-CoV E protein PBM as a virulence determinant *in vivo*. Infection with recombinant viruses lacking an E protein PBM were attenuated in mice, which was accompanied by a decreased expression of inflammatory cytokines during infection, and a substantial increase of survival. In contrast, all mice infected with viruses containing E protein PBM died. We further found that the E protein PBM interacted with the cellular protein syntenin during SARS-CoV infection, affecting p38 mitogen-activated protein kinase (MAPK) activation, a protein involved in the expression of inflammatory cytokines, responsible of the pathogenicity associated to SARS-CoV infection. In addition, mice treated with a p38 MAPK inhibitor showed a significant increase in survival after infection with SARS-CoV. Together, our findings provide novel insights into how highly pathogenic viruses, such as SARS-CoV, induce virulence, suggesting potential therapeutic targets to improve the prognosis in patients.

## Results

### Generation of recombinant SARS-CoVs lacking E protein PBM

**Figure 1:**
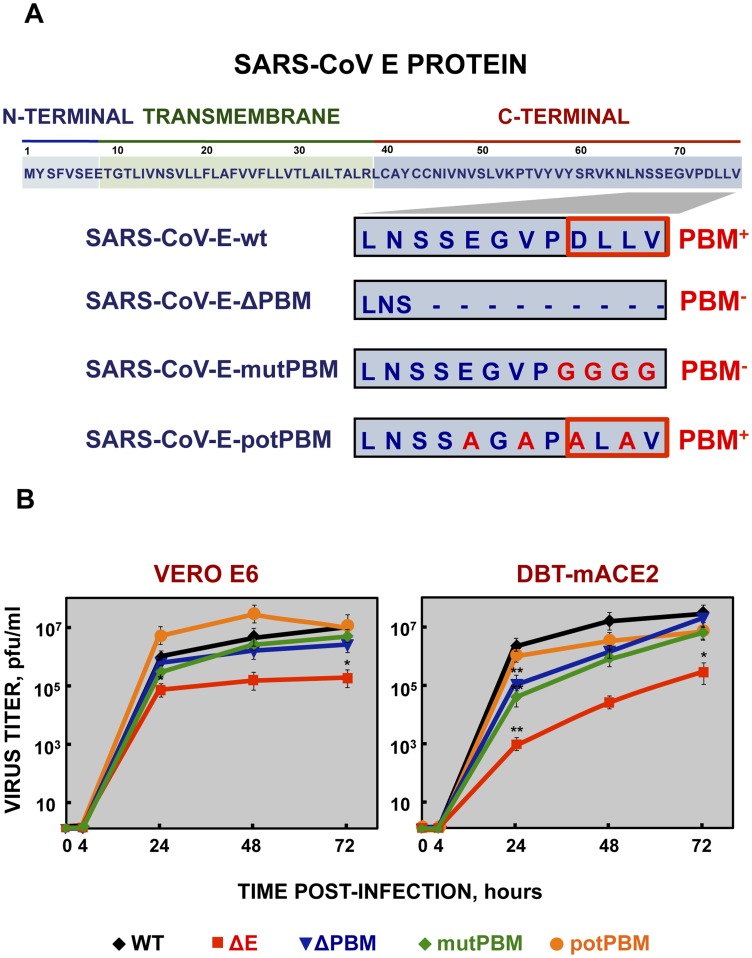
Generation of recombinant SARS-CoVs with E protein PBM truncated or mutated by reverse genetics and growth kinetics in cell culture. (A) Top, representation of SARS-CoV E protein sequence and its corresponding domains. Below, sequences corresponding to the end of E protein are shown in boxes for the different viruses. SARS-CoV-E-wt represents the wild type sequence. In SARS-CoV-E-ΔPBM and SARS-CoV-E-mutPBM, E protein PBM was eliminated by deletion or point mutations, reducing or maintaining the full-length protein, respectively. In SARS-CoV-E-potPBM, four amino acids of E protein were replaced to alanine, to generate a non-native new potential PBM. PBM^+^ and PBM^−^ represent the presence or absence of a PBM within E protein sequence, respectively. Red boxes highlight PBMs within E protein. (B) Subconfluent monolayers of Vero E6 and DBT-mACE2 cells were infected with wt, ΔE, ΔPBM, mutPBM and potPBM viruses at an MOI of 0.05. Culture supernatants collected at 4, 24, 48 and 72 hpi were titrated by plaque assay. Error bars represent standard deviations of the mean of results from three experiments. Statistically significant data are indicated with one (*P*<0.05) or two (*P*<0.01) asterisks.

To evaluate the role of SARS-CoV E protein PBM in virus pathogenesis, a set of recombinant SARS-CoVs with E protein PBM mutated or truncated (SARS-CoV-E-PBMs) were generated using an infectious cDNA encoding a mouse adapted (MA15) SARS-CoV [31], [32]. Infection of BALB/c mice with SARS-CoV-MA15 causes morbidity, mortality and pulmonary pathology, similar to the symptoms observed in human SARS [31]. In SARS-CoV-E-ΔPBM, abbreviated as ΔPBM, the last 9 amino acids of E protein were deleted, truncating the carboxy-terminus, and eliminating the PBM (Figure 1A). In contrast, the PBM was abolished in SARS-CoV-E-mutPBM (mutPBM) by mutating the last 4 amino acids to glycine, maintaining the full-length SARS-CoV E protein. In the last recombinant, termed SARS-CoV-E-potPBM (potPBM), four amino acids within E protein were replaced by alanine, modifying the E protein carboxy-terminal sequence while maintain the consensus PBM residues (Figure 1A).

To test whether mutation or deletion of SARS-CoV E protein PBM alters virus fitness *in vitro*, growth kinetics of SARS-CoV-E-PBM mutants were analyzed in comparison to the parental virus (wt) and the virus lacking the full-length E protein SARS-CoV-ΔE (ΔE) in monkey Vero E6 and mouse DBT-mACE2 cells [33] (Figure 1B). Despite the observed replication defects of the ΔPBM and mutPBM viruses at 24 hpi in DBT-mACE2 cells, the parental virus including native E protein or mutants lacking a PBM reached similar titers at 72 hpi, both in Vero E6 cells and in DBT-mACE2 cells (Figure 1B). In contrast, the titer of ΔE virus was reduced around 50-fold (Figure 1B). This result indicated that SARS-CoV E protein PBM was not essential for efficient virus growth in Vero E6 cells and, at late times post infection, in DBT-mACE2 cells.

### Pathogenicity of SARS-CoV E protein PBM mutants in BALB/c mice

**Figure 2:**
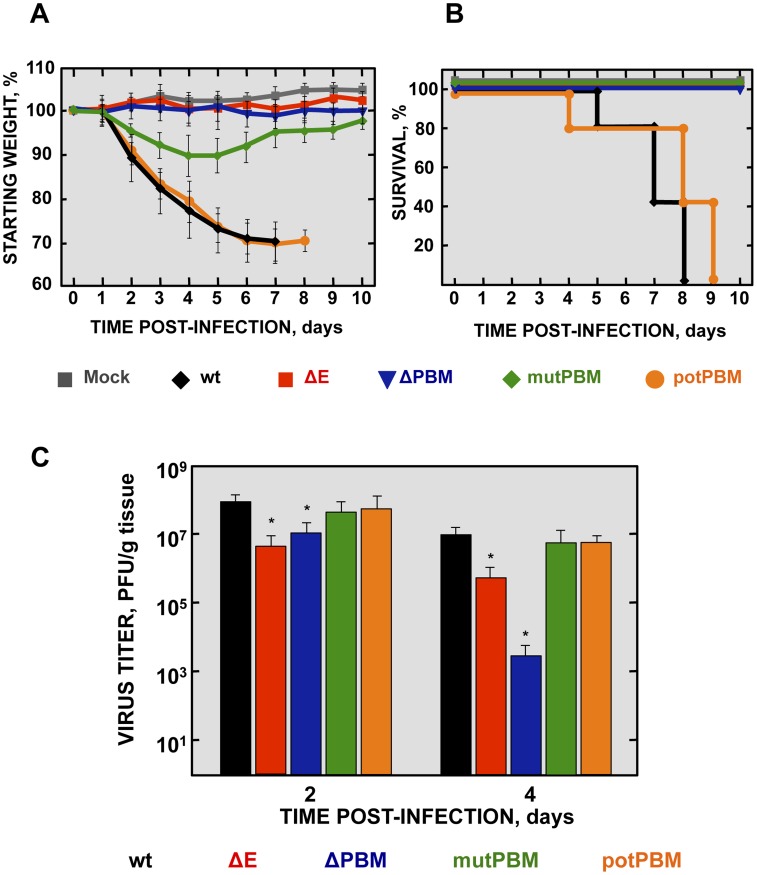
Virulence and viral growth of SARS-CoV-E-PBM-infected mice. 16-week-old BALB/c mice were intranasally inoculated with 100,000 pfu of wt, ΔE, ΔPBM, mutPBM and potPBM viruses. Weight loss (A) and survival (B) were monitored for 10 days. Data represent two independent experiments with 5 mice per group. Differences in weight loss between attenuated and virulent viruses were statistically significant (*P*<0.01). (C) Viral titer in lungs was determined at 2 and 4 days post infection (n = 3, each day). Error bars represent standard deviations. Statistically significant data are indicated with one (*P*<0.05) asterisk.

To analyze the pathogenicity of SARS-CoV-E-PBM mutants, 16 week-old female BALB/c mice were intranasally inoculated with recombinant viruses. Body weight (Figure 2A) and mortality (Figure 2B) of each mouse were monitored daily.

Mock-infected mice and those infected with a virus lacking E protein, did not lose weight and all survived. In contrast, mice infected with recombinant viruses including an E protein PBM, either the original PBM (wt) or a potential PBM (potPBM), underwent severe weight loss (Figure 2A) and developed signs of illness, including shaking, ruffling of the fur, and loss of mobility, resulting in 100% mortality by 9 days post infection (dpi) (Figure 2B). Interestingly, mice infected with the viruses in which E protein PBM was abolished (mutPBM) or deleted (ΔPBM), showed moderate or no weight losses, respectively, and 100% survival in both cases (Figure 2B). The fact that ΔPBM is more attenuated that mutPBM suggests the presence of sequences, outside PBM, further contributing to virus pathogenesis. These results indicated that elimination of E protein PBM led to virus attenuation *in vivo* and that the presence of a functional PBM conferred virulence, as mutant potPBM in which 4 amino acids in the carboxy-terminal domain were mutated to alanine, conserving consensus residues in the PBM was still virulent.

To evaluate the effect of the E protein PBM in virus growth *in vivo*, BALB/c mice were intranasally inoculated with recombinant viruses, and viral titers in the lung were determined at 2 and 4 dpi (Figure 2C). Viruses ΔE and ΔPBM showed decreased titers in lungs at both 2 and 4 dpi, as compared with the wt or potPBM viruses. Mutant ΔE replicated at a higher level than ΔPBM at 4 dpi in lungs of infected mice, suggesting that the ΔPBM E protein may induce an antiviral state after infection. Interestingly, the virus lacking a functional E protein PBM but conserving a full-length E protein (mutPBM) grew to similar levels as the wt virus at 2 and 4 dpi, indicating that a virus lacking a canonical PBM in E protein, but conserving E protein full-length displayed an attenuated phenotype, although it efficiently replicated *in vivo*.

### Lung pathology in mice infected with SARS-CoV E protein PBM mutants

**Figure 3:**
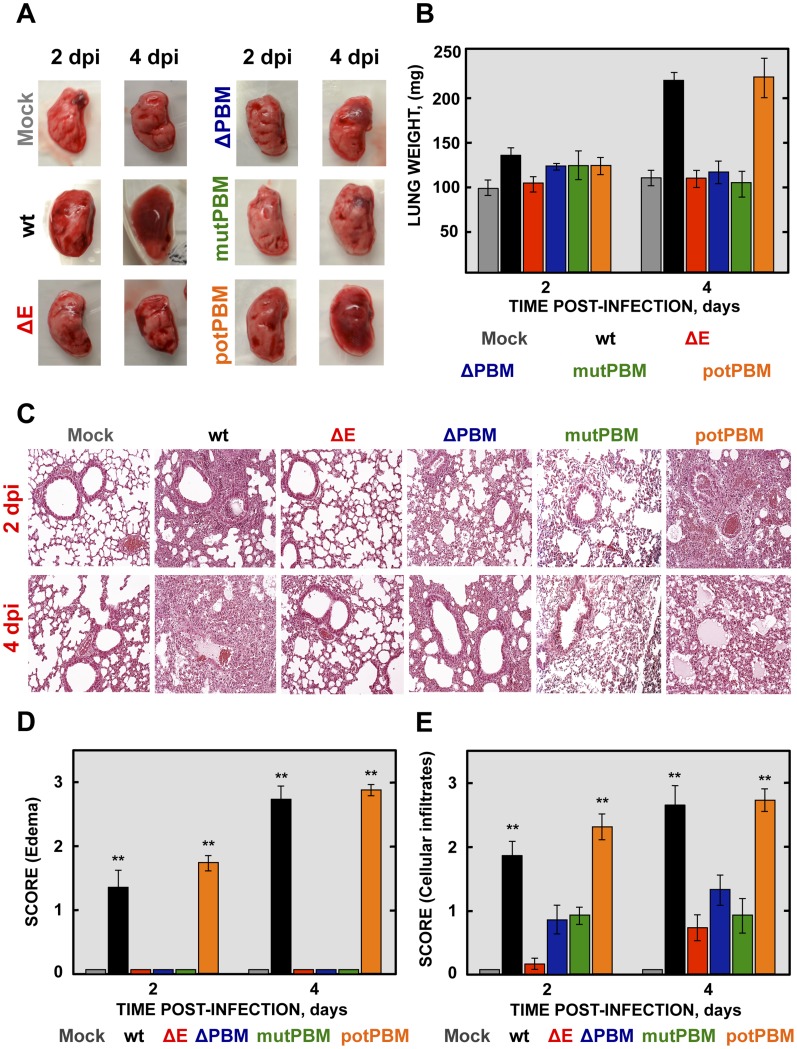
Lung pathology of mice infected with recombinant SARS-CoV-E-PBM mutants. 16-week-old BALB/c mice were inoculated intranasally with 100,000 pfu of wt, ΔE, ΔPBM, mutPBM and potPBM viruses. (A) Gross pathology of mouse lungs infected with recombinant viruses at 2 and 4 dpi. (B) Weight of left lungs excised from infected mice sacrificed at the indicated days (n = 3, each day). Error bars represent standard deviations. (C) Lung tissue sections from mice infected with recombinant viruses were prepared at 2 and 4 dpi and stained with hematoxylin and eosin. Three independent mice per group were analyzed. (D and E) Pathology scoring in lung of mice infected with SARS-CoV recombinant mutants. Lungs were harvested at 2 and 4 days and scored in a blinded fashion using 3 mice per condition on a scale of 0 (none) to 3 (severe), estimated according to previously described procedures [34]. Data are presented for edema (D) and cellular infiltrates (E). Mean values are reported and statistically significant data are indicated with two (*P*<0.01) asterisks. Original magnification was 20x. Representative images are shown.

To analyze the mechanisms by which E protein PBM confers virulence *in vivo*, lung pathology was examined in infected BALB/c mice at 2 and 4 dpi. At these time points, no obvious gross lesions or changes in weight were observed in the lungs of non-infected mice or in those from mice infected with viruses lacking functional E protein PBM (ΔE, ΔPBM and mutPBM). In contrast, at 2 and especially at 4 dpi, lungs of mice infected with SARS-CoVs with an E protein containing a functional PBM (wt and potPBM) were highly edematous, with profuse hemorrhagic areas, leading to significant lung weight increase at 4 dpi, probably due to leukocyte infiltration (Figures 3A and 3B).

To further characterize the pathology induced in BALB/c mice by the infection with SARS-CoVs with and without E protein PBM, lung sections were collected at 2 and 4 dpi, stained with hematoxylin and eosin (Figure 3C) and pulmonary histopathology scores for edema and cellular infiltrates were quantified according to previously described procedures [34] (Figures 3D and 3E). Histological examination of lungs from mock or SARS-CoV-ΔE-infected mice showed minimal evidence of damage or cellular infiltration at 2 and 4 dpi (Figure 3C, 3D and 3E). In contrast, mice infected with recombinant viruses containing functional E protein PBM (wt and potPBM) revealed interstitial and peribronchial cell infiltration and edema in both alveolar and bronchiolar airways at 2 and, mainly, at 4 dpi (Figure 3C, 3D and 3E). Interestingly, mice infected with viruses containing E protein but lacking functional PBM sequences (ΔPBM and mutPBM), showed minimal epithelial damage or lung edema and only small amounts of inflammatory cell infiltrates at 4 dpi (Figure 3C, 3D and 3E). These data indicated that the attenuation observed for viruses lacking E protein PBM correlated with decreased lung pathology.

### Effect of SARS-CoV E protein PBM on host gene expression

**Figure 4:**
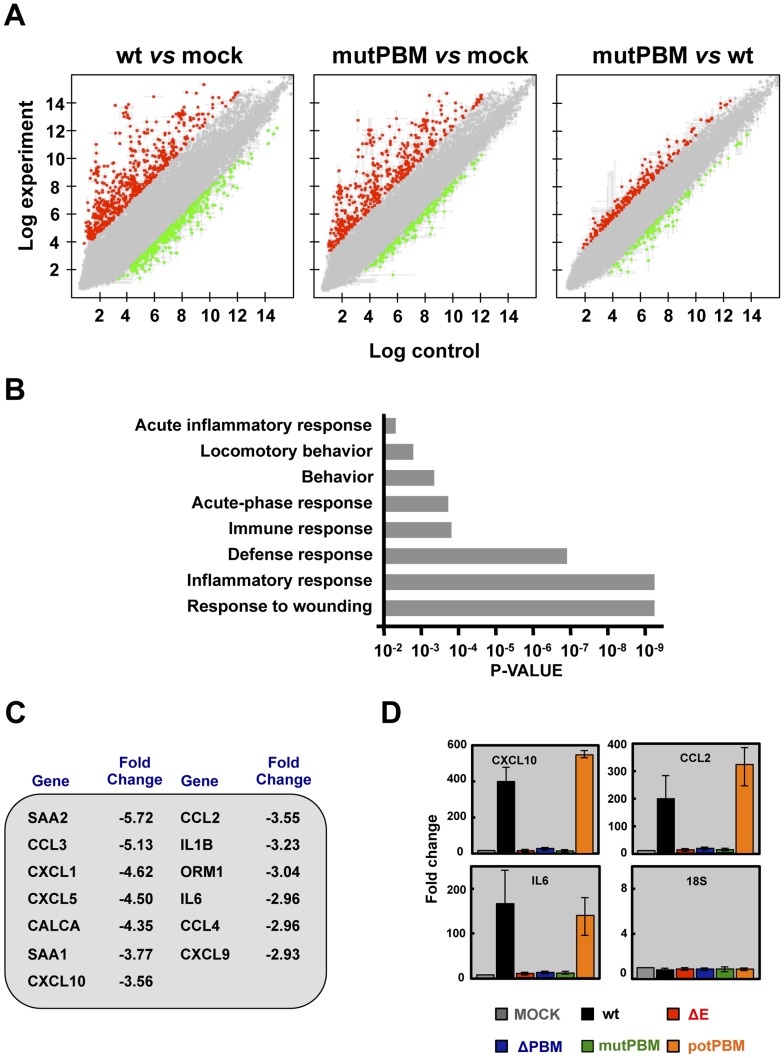
Effect of SARS-CoV E protein PBM on host gene expression. (A) Comparison of gene expression in lungs of infected mice using microarrays: wt versus mock-infected, mutPBM versus mock-infected and mutPBM versus wt-infected mice. Red spots indicate upregulated gene transcripts (fold change, >2) and green spots indicate downregulated gene transcripts (fold change, <−2). Only genes with a FDR of <0.01 were considered as candidate genes. (B) Candidate genes that were downregulated in mutPBM infected mice compared wt infected ones, were grouped on Gene Ontology terms. Numbers on the *x* axis indicate DAVID FDR values. (C) Selection of differentially expressed genes found in at least one functional group using DAVID software. The numbers indicate the fold change for each gene in mutPBM versus wt-infected mice. (D) Expression of inflammatory cytokines evaluated by RT-qPCR. Three independent experiments were analyzed with similar results in all cases. Error bars represent standard deviations of the mean of results from three experiments.

The effect of the presence of a PBM in SARS-CoV E protein on host gene expression during BALB/c mice infection was analyzed using microarrays at 2 dpi. MIAME-compliant results of the microarrays have been deposited in the Gene Expression Omnibus database (GEO [National Center for Biotechnology Information], accession code GSE52920). A total of 922 and 640 cellular genes were differentially expressed in lung of mice infected with SARS-CoV with (wt) or without functional E protein PBM (mutPBM) as compared with mock-infected mice (Figure 4A). Remarkably, 319 genes were differentially expressed in mice infected with mutPBM compared to wt, despite a difference at only 4 amino acid positions between the two viruses. Of these, 218 genes were found to be upregulated and 101 genes were downregulated (Figure 4A). Interestingly, analysis using DAVID software [35] revealed that most of the downregulated genes in mutPBM versus wt infections clustered within wound response and inflammatory and defense response pathways (Figure 4B). The most significant genes present in at least one of these groups were serum amyloid A2 (*SAA2*), chemokine (C-C motif) ligand 3 (*CCL3*), chemokine (C-X-C motif) ligand 1 (*CXCL1*), chemokine (C-X-C motif) ligand 5 (*CXCL5*), calcitonin (*CALCA*), serum amyloid A1 (*SAA1*), chemokine (C-X-C motif) ligand 10 (*CXCL10*), chemokine (C-C motif) ligand 2 (*CCL2*), interleukin 1 beta (*IL1B*), orosomucoid 1 (*ORM1*), interleukin 6 (*IL6*), chemokine (C-C motif) ligand 4 (*CCL4*) and chemokine (C-X-C motif) ligand 9 (*CXCL9*) (Figure 4C). The differential expression of a group of cellular genes identified in the microarray (*CXCL10*, *CCL2* and *IL6*) was confirmed by RT-qPCR analysis using RNA from the lungs of mice infected with all the recombinant viruses generated, collected at 2 dpi, in relation to RNAs from mock-infected mice. *18S* ribosomal RNA (rRNA) was used to normalize the data [36], [37] (Figure 4D). Using both microarray and RT-qPCR, we identified genes differentially expressed in the lungs of mice infected with viruses with or without E protein PBM (Figures 4C and 4D). Viruses lacking E protein PBM induced a decreased expression of inflammatory cytokines. These data indicated that the exacerbated host innate immune response triggered during SARS-CoV infection was reduced in the absence of SARS-CoV E protein PBM, which may explain the attenuated phenotype of these viruses.

### Identification of cellular factors interacting with SARS-CoV E protein

**Figure 5:**
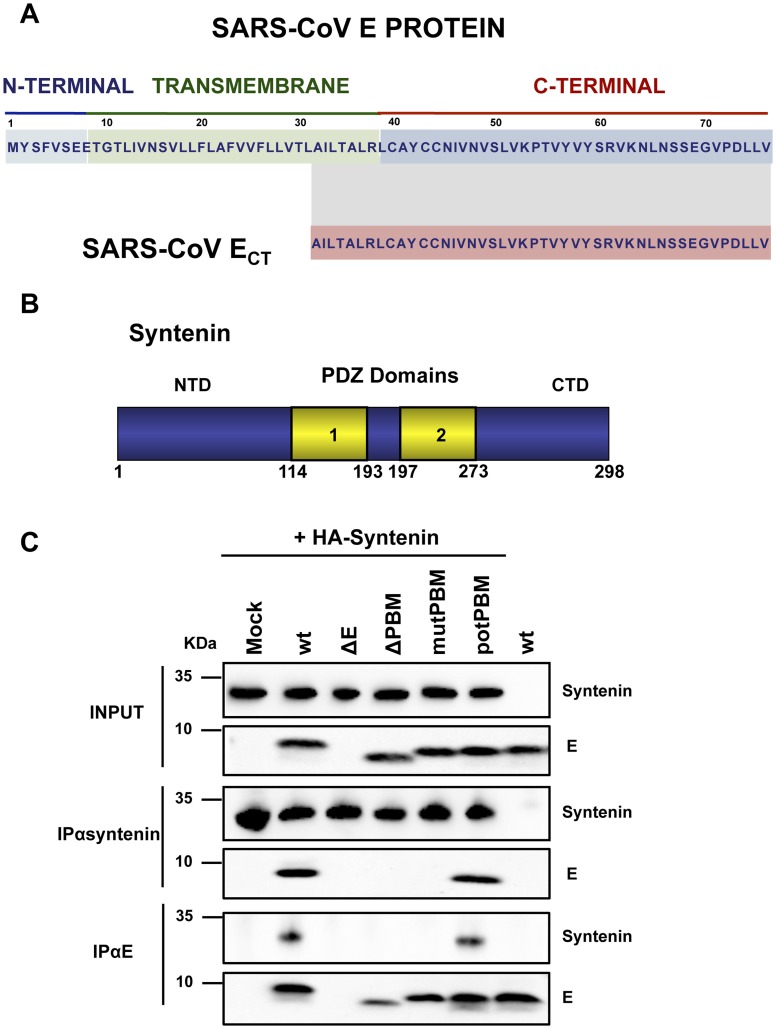
Interaction of SARS-CoV E protein with cellular syntenin. (A) Sequence of SARS-CoV E protein and the region containing amino acids 36–76 (SARS-CoV E_CT_) that was used as bait for the yeast two-hybrid screening. (B) Schematic representation of syntenin. Numbers at the bottom indicate the amino acids at the beginning and the end of the different domains. NTD; N-terminal domain; CTD, C-terminal domain; PDZ 1 and 2, PDZ domains. (C) Vero E6 cells transfected with a plasmid encoding an N-terminal HA-tagged syntenin were mock-infected (mock) or infected with recombinant viruses containing (wt and potPBM) or lacking (ΔE, PBM and mutPBM) E protein PBM, respectively. As a control, mock-transfected cells were infected with the wt virus (wt, last lane). Cells were lysed and subjected to immunoprecipitation using a monoclonal anti-HA antibody or polyclonal anti-E antibody to pulldown syntenin or E protein, respectively. The presence of E and syntenin proteins was analyzed in the precipitated fractions.

SARS-CoVs defective in E protein PBM presented an attenuated phenotype that correlated with a decreased inflammatory response. The absence of this motif may likely imply changes in interaction patterns with cellular proteins that may explain their reduced virulence. To identify these cellular factors, a yeast two-hybrid screen was performed using the carboxy-terminal domain of SARS-CoV E protein, where amino acids 36–76 of E protein carboxy-terminus (E_CT_) were used as bait (Figure 5A). A random-primed cDNA library from human lung was screened. One of the most prominent results of the screening was the interaction between E_CT_ and the syndecan binding protein (syntenin) (Figure 5B), with a total of 13 positive clones corresponding to this protein (GenBank accession number NM_005625.3). The interaction was classified with a high confidence score (predicted biological score of B) [38]. Syntenin is a 32 kDa protein composed of a 113 amino acid N-terminal domain (NTD) with no obvious structural motifs, followed by two adjacent tandem PDZ domains (PDZ1 and PDZ2), that could mediate its interaction with E protein and a short 24 amino acid C-terminal domain (CTD) [39] (Figure 5B). Interestingly, all 13 recovered syntenin cDNAs interacting with the E protein carboxy-terminus identified in the yeast two-hybrid platform contained the same two PDZ domains present in the cellular syntenin.

To determine whether the cellular protein syntenin is also associated with SARS-CoV E protein in infected cells, and whether this interaction was mediated through the E protein PBM, Vero E6 cells were transfected with a plasmid encoding an N-terminal HA-tagged syntenin and then infected with recombinant viruses with or without E protein PBM. Non-transfected cells infected with wt virus were used as control. Syntenin or E protein were immunoprecipitated using extracts of infected cells using an HA specific monoclonal antibody or an E protein polyclonal antibody, respectively. Inmunoblot analysis using E and HA specific antibodies revealed that E protein coprecipitated with syntenin in cells infected with recombinant viruses containing the wt or potential E protein PBM (Figure 5C). In contrast, E protein did not coprecipitate with syntenin in cells infected with viruses lacking the E protein or its PBM (Figure 5C). These results indicate that the last 4 amino acids of SARS-CoV E protein form a functional PBM that, in the context of virus infection, mediate its association with the cellular protein syntenin. The absence of this interaction could be playing a role in the attenuation observed in viruses lacking E protein PBM.

### Colocalization of SARS-CoV E protein and syntenin in infected-cells and in cells transfected with a plasmid expressing E protein

**Figure 6:**
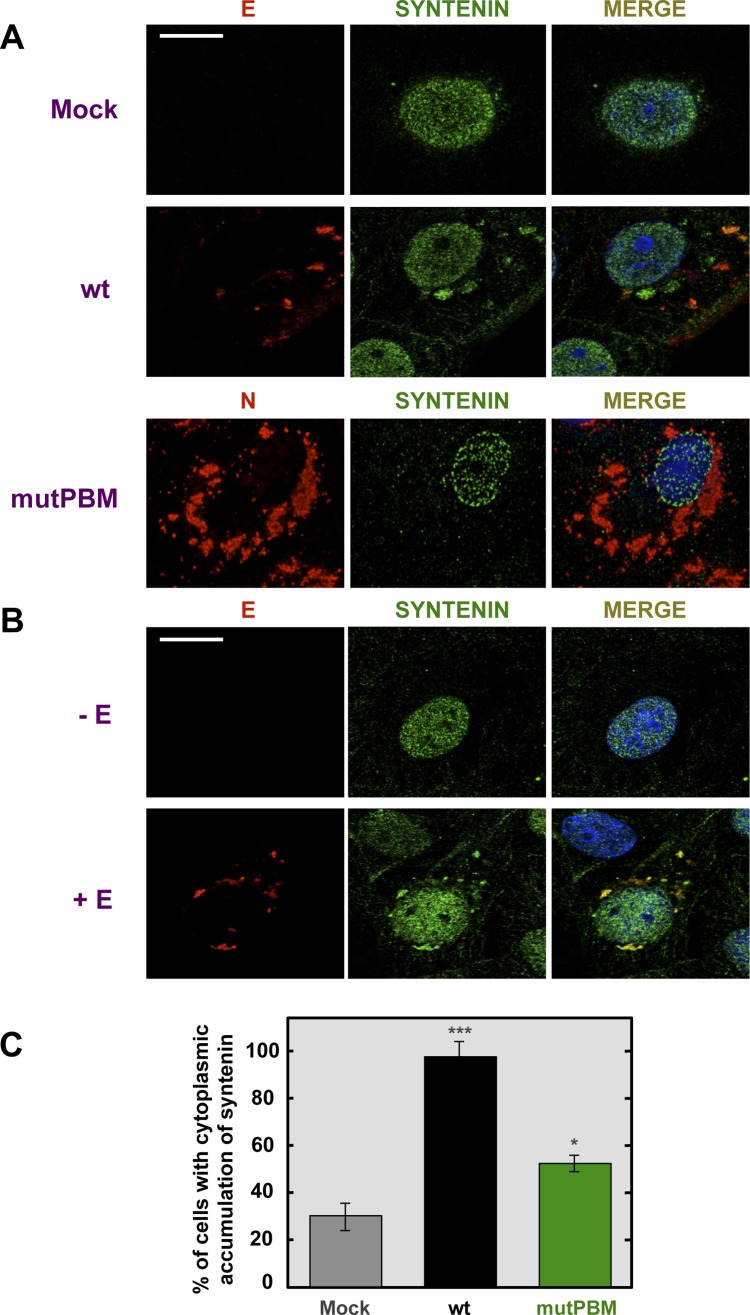
Colocalization of SARS-CoV E protein and syntenin in transfected and infected cells. Vero E6 were mock-infected or infected with the wt virus at an MOI of 0.3 (A) or transfected with an empty plasmid (−E) or a plasmid expressing SARS-CoV E protein (+E) (B). At 24 hpi and 24 hours post transfection (hpt) for (A) and (B), respectively, cells were fixed with 4% paraformaldehyde and E or N proteins (red) and syntenin (green) were labeled with specific antibodies, nuclei were stained with DAPI (blue). Areas of colocalization of the two proteins appear yellow in the merged images. Scale bar = 10 µm. (C) Percentage of cells showing a cytoplasmic accumulation of syntenin after mock-infection or infected with wt or mutPBM viruses (n>50). Statistically significant data are indicated with one (*P*<0.05) or three (*P*<0.001) asterisks.

To evaluate whether SARS-CoV E protein and syntenin colocalize during infection and to determine if syntenin localization was altered during SARS-CoV infection, mock-infected Vero E6 cells and cells infected with the wt or mutPBM virus were analyzed by confocal immunomicroscopy using specific antibodies against the cellular protein syntenin and the SARS-CoV nucleocapsid (N) and E proteins (Figure 6A). Syntenin was predominantly present in the nucleus of mock-infected cells. Upon wt infection, E protein was mainly localized at perinuclear regions as previously described [15]. Interestingly, after infection, syntenin partially colocalized with E protein in the perinuclear region and also relocalized to locations close to the plasma membrane (Figure 6A). Furthermore, infection with the mutPBM virus led to N protein cytoplasmic localization as previously described [40], and to a decrease in the relocalization of syntenin to the cytoplasm as compared with the parental virus.

To determine whether E protein was involved in syntenin relocalization during SARS-CoV infection, Vero E6 cells were transiently transfected with an empty plasmid or a plasmid expressing E protein and both, syntenin and E protein, were detected with specific antibodies (Figure 6B). As previously described with mock-infected cells, syntenin was mainly detected in the nucleus of cells transfected with an empty plasmid. However, in cells transfected with the plasmid expressing E protein, syntenin colocalized with E protein in the perinuclear region and adopted a distribution close to the plasma membrane (Figure 6B). Furthermore, the percentage of mock-infected versus virus-infected cells that showed cytoplasmic accumulation of syntenin was quantified (Figure 6C). Syntenin accumulated in the cytoplasm of 98.5% of the cells infected with the parental virus, whereas 31.2% of the mock-infected cells displayed syntenin in the cytoplasm. In cells infected with the mutPBM virus only 51.5% showed syntenin in the cytoplasm. These results indicated that syntenin partially colocalized with SARS-CoV E protein, and that it was redistributed from the nucleus to perinuclear regions, where E protein is accumulated, and also to regions close to the plasma membrane.

### p38 MAPK activation in the lungs of mice infected with recombinant SARS-CoV E protein PBM mutants

**Figure 7:**
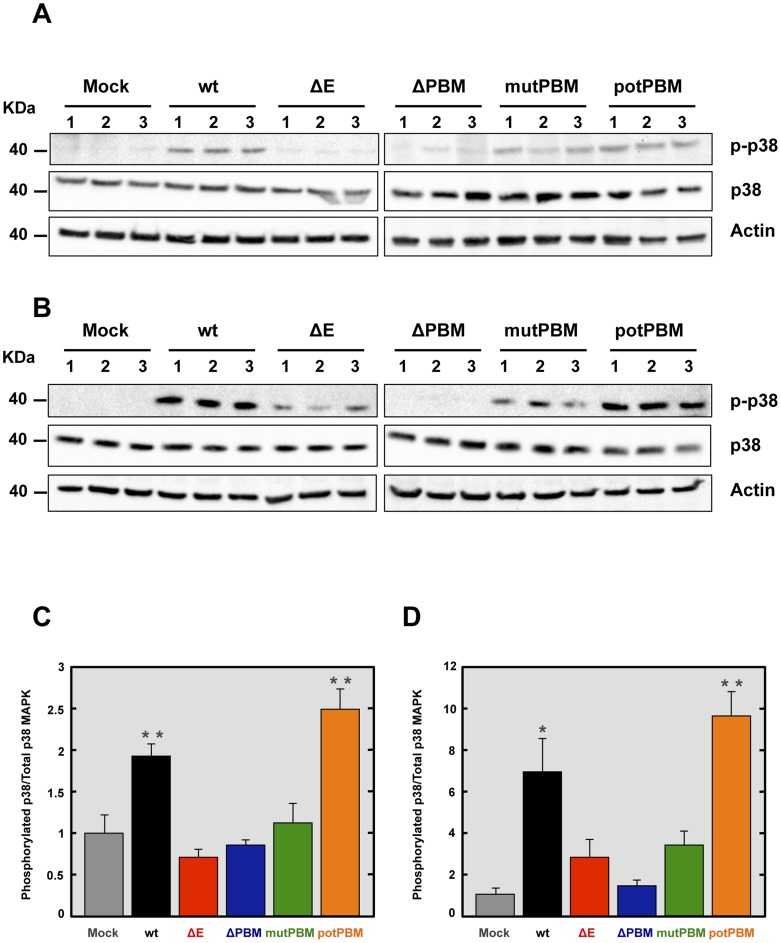
Activation of p38 MAPK in SARS-CoV-E-PBM mutants infected mice and cells. Lung proteins were extracted from infected mice at 2 dpi. (A) The active phosphorylated (p-p38) and total (p38) p38 MAPK in lungs of three infected mice per condition were evaluated by Western blot analysis. (B) The active phosphorylated (p-p38) and total (p38) p38 MAPK in Vero E6 infected cells were evaluated by Western blot analysis. (C and D) Phospho and total p38 MAPK amounts were quantified by densitometric analysis. The graph shows the phosphorylated p38/total p38 MAPK ratio in wt, ΔE, ΔPBM, mutPBM and potPBM infected mice at 2 dpi (C) or Vero E6 cells at 24 hpi (D). Error bars represent the means of three mice analyzed for each condition. Statistically significant data are indicated with one (*P*<0.05) or two (*P*<0.01) asterisks.

Syntenin has been described as an important scaffolding protein that can initiate a signaling cascade resulting in the induction of p38 MAPK [41]. In this model, after its interaction with the extracellular matrix, syntenin induces phosphorylation and therefore, activation of p38 MAPK, a protein involved in the expression of proinflammatory cytokines [42], [43]. To determine whether p38 MAPK was differentially activated in the lungs of mice infected with recombinant SARS-CoV with or without E protein PBM, 16 week-old female BALB/c mice were intranasally inoculated with these viruses. The activation of p38 MAPK was studied by Western blot analysis at 2 dpi, using a phospho-p38 MAPK (p-p38) specific antibody to detect the active form, and an antibody specifically recognizing the total endogenous p38 MAPK. Actin served as loading control. Interestingly, the levels of active p38 MAPK were increased in the lungs of mice infected with SARS-CoV containing E protein PBM, compared to those found in lungs of mice infected with viruses lacking E protein PBM (Figures 7A and 7C). To reinforce the data, p38 MAPK activation was studied in infected cells. To this end, Vero E6 cells were mock-infected or infected with recombinant viruses with an E protein with or without a PBM. Then, p38 MAPK activation was analyzed by Western blot at 24 hpi. Interestingly, an increase in p38 MAPK activation was observed during infection with viruses containing E protein PBM, similarly to what was observed in the lungs of SARS-CoV-infected mice (Figures 7B and 7D). These results indicated that the E protein PBM is involved in p38 MAPK activation in response to SARS-CoV infection.

### Role of syntenin in the E protein PBM-dependent p38 MAPK activation

**Figure 8:**
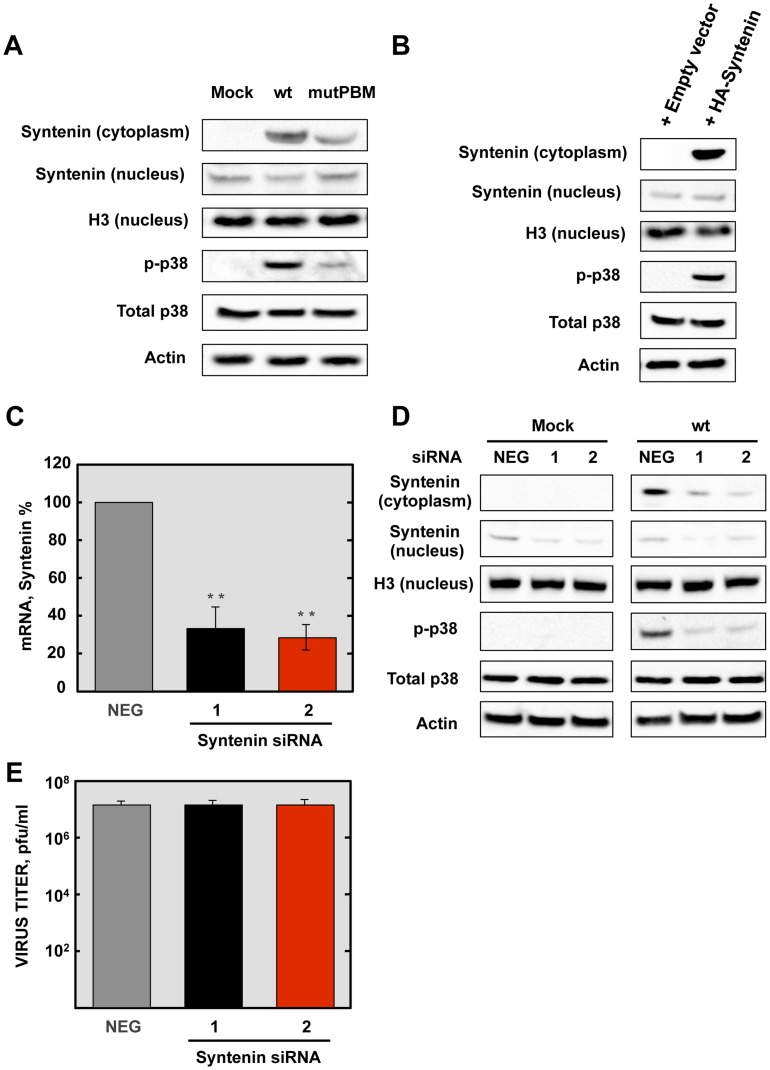
Role of syntenin in the E protein PBM-dependent p38 MAPK activation. (A) Vero E6 cells were mock-infected or infected with recombinant viruses containing (wt) or lacking (mutPBM) E protein PBM, and the presence of syntenin in the cytoplasm and nucleus and active p38 MAPK (p-p38) were detected by Western blot analysis at 24 hpi. As controls, histone H3 (H3), total p38 MAPK (Total p38) and actin were analyzed. (B) Vero E6 cells were transfected with an empty plasmid (empty vector) or a plasmid encoding a HA-tagged syntenin (HA-syntenin), and the presence of syntenin in the cytoplasm and nucleus, and active p38 MAPK were detected by Western blot analysis at 24 hpt. As controls, histone H3, total p38 MAPK, and actin were analyzed. (C) Quantification by qRT-PCR of syntenin mRNA in cells transfected with syntenin-specific siRNA (1 and 2) compared to reference levels from cells transfected with a validated-negative control siRNA (NEG). Mean values are reported, and statistically significant data are indicated with two (*P*<0.01) asterisks. (D) Effect of silencing syntenin expression on Vero E6 cells, mock-infected or infected with the wt virus. The presence of syntenin in the cytoplasm and nucleus and active p38 MAPK were detected by Western blot at 24 hpi. As controls, histone H3, total p38 MAPK and actin were analyzed. (E) Viral titers of wt virus in syntenin silenced Vero E6 cells were determined at 24 hpi. The experiments were performed three times, and the data represent the averages of triplicates. Standard deviations are indicated as error bars. hpi. The experiments were performed three times, and the data represent the averages of triplicates. Standard deviations are indicated as error bars.

We have shown above that SARS-CoV E protein PBM interacted with syntenin, and that infection with SARS-CoVs containing an E protein with a functional PBM led to an increase in p38 MAPK activation. As syntenin promotes p38 MAPK activation [41], we hypothesized that syntenin relocalization from nucleus to cytoplasm during infection with SARS-CoV, containing an E protein including the PBM, may be responsible for the activation of the p38 MAPK pathway. To test this hypothesis, Vero E6 cells were mock-infected or infected with recombinant SARS-CoVs including an E protein with (wt) or without (mutPBM) E protein PBM. At 24 hpi, the cytosolic and nuclear fractions from SARS-CoV infected cells were collected, and the levels of syntenin and extent p38 MAPK activation in both fractions were determined by Western blot analysis using specific antibodies for syntenin and the non-phosphorylated and phosphorylated forms of p38 MAPK. The levels of histone H3, total p38 MAPK and actin were used as controls. Syntenin levels in the cytosol fraction were increased during wt infection. Interestingly, mutPBM virus retained the ability to mislocalize a substantial amount of syntenin to the cytoplasmic fraction, possibly due to the ability of E protein to bind SARS-CoV 3a protein, which also contains a PBM, in addition to the one present in the E protein [44], [45]. Furthermore, the presence of syntenin in the cytosol correlated with the activation of p38 MAPK (Figure 8A). To determine whether syntenin relocalization from nucleus to cytoplasm mediated p38 MAPK activation, Vero E6 cells were transfected with an empty plasmid or a plasmid expressing human syntenin, and presence of this protein in the nucleus or the cytoplasm of the infected cells and the levels of p38 MAPK phosphorylation were studied by Western blot analysis using specific antibodies. The levels of histone H3, total p38 MAPK and actin were used as controls. The results showed the presence of syntenin in the nucleus of cells transfected with both plasmids. In contrast, syntenin was only detected in the cytoplasmic fraction when the syntenin was overexpressed, and failed to accumulate in the nuclear fraction. This observation could be explained by the previously reported saturation of the nuclear import machinery, which leads to cytoplasmic retention of overexpressed proteins, as described for other proteins [46]. Interestingly, the presence of syntenin in the cytoplasm correlated with an increase of p38 MAPK activation (Figure 8B).

To further confirm the role of syntenin in the activation of p38 MAPK during infection by SARS-CoV with an E protein containing a functional PBM, siRNAs specifically designed to inhibit syntenin expression were used in mock-infected cells or in cells infected with the parental virus. p38 MAPK activation was analyzed by Western blot. Vero E6 cells were transfected twice by reverse transfection with either 25 nM of a validated negative control siRNA (NEG) or with similar amounts of each of two different siRNAs targeting endogenous syntenin. At 24 hpt, cells were mock-infected or infected with the parental virus at an MOI of 0.3. At 24 hpi, syntenin mRNA levels were significantly (60 to 70%) reduced in syntenin-silenced cells in relation to the cells transfected with a validated negative-control siRNA, as determined by qRT-PCR using specific Taqman gene expression assays (Figure 8C). Accordingly, syntenin levels evaluated by Western blot analysis were also significantly reduced in syntenin-silenced cells. Moreover, this silencing was found to have a higher apparent impact on the reduction of this protein in the cytoplasm, probably because that this protein accumulates more efficiently in the nucleus than the cytoplasm after protein expression. Interestingly, inhibition of syntenin expression was accompanied by a decreased in p38 MAPK activation during the infection with the parental virus (Figure 8D), whereas no changes in virus titers were observed (Figure 8E). Overall, these results support the hypothesis that the interaction of E protein PBM with syntenin facilitates the recruitment of syntenin in the cytosol and leads to p38 MAPK activation.

### Effect of a p38 MAPK inhibitor on the survival of rSARS-CoV-MA15-infected mice

**Figure 9:**
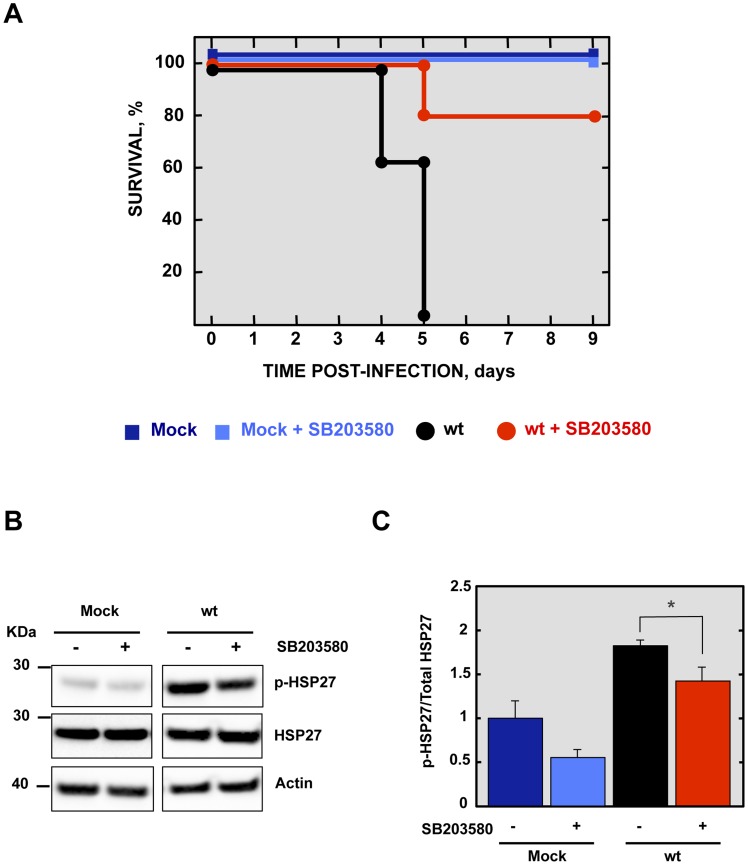
Effect of p38 MAPK inhibitor in rSARS-CoV-MA15-infected mice. 16-week-old BALB/c mice were mock-infected or inoculated intranasally with 100,000 pfu of wt virus. At 4 hpi and every 12 h from days 1 to 8, mock pfu of wt virus. At 4 hpi and every 12 h from days 1 to 8, mock hpi and every 12 h from days 1 to 8, mock h from days 1 to 8, mock-infected and wt-infected mice were intraperitoneally injected with SB203580 (6 mg mg/kg of body weight/day). (A) Animals were monitored daily for mortality. Data represent three independent experiments with 5 mice per group. (B) The active phosphorylated (p-HSP27) and total HSP27 in lungs of three infected mice per condition were evaluated by Western blot analysis. (C) Phospho and total HSP27 amounts were quantified by densitometric analysis. The graph shows the phosphorylated HSP27/total HSP27 ratio at 2 dpi in lungs of mock dpi in lungs of mock-infected mice or mice infected with the parental virus, treated or not with SB203580. Error bars represent the means of three mice analyzed for each condition. Statistically significant data are indicated with one (*P*<0.05) asterisk.

To analyze the contribution of SARS-CoV E protein PBM-mediated p38 MAPK activation to the disease observed during SARS-CoV infection in mice, 16 week-old female BALB/c mice were intraperitoneally administered with a control buffer or SB203580, a highly specific inhibitor of p38 MAPK, and were mock-infected or infected with the rSARS-CoV-MA15 virus. Mock-infected mice treated with the inhibitor showed 100% survival and no signs of clinical disease (Figure 9A). Interestingly, in the case of mice treated with the p38 MAPK inhibitor and infected with the parental virus, survival increased to 80% as compared with non-treated mice, with a mortality of 100%. SB203580 inhibits p38 MAPK catalytic activity by binding to the ATP-binding pocket, blocking the activation of several proteins regulated by the p38 MAPK pathway, including the heat-shock protein 27 (HSP27) [47], but does not inhibit phosphorylation of p38 MAPK by upstream kinases [48]. Therefore, to analyze whether SB203580 actually reduced p38 MAPK activity in lung tissue of virus-infected mice, the activation of HSP27 was studied by Western blot analysis at 2 dpi, using a phospho-HSP27 (p-HSP27) specific antibody to detect the active form, and an antibody specifically recognizing the total endogenous HSP27. Actin served as loading control. Results showed that the levels of active HSP27 were significantly reduced in the lungs of mice treated with SB203580 and infected with the parental virus, compared to those that were infected and non-treated (Figures 9B and 9C), indicating that p38 MAPK activity was diminished by SB203580. These results indicated that p38 MAPK activation is an important factor for SARS-CoV-induced disease.

## Discussion

Cellular factors containing PDZ domains participate in a complex network of protein-protein interactions that modulate many diverse biological processes such as cell polarity, cell-cell interactions, control of proliferation, migration, immune cell recognition and signal transduction pathways [28], [49], [50]. Alterations of these highly regulated processes can lead to important disorders, including several types of cancer [51]. Viruses have evolved proteins containing PBM to exploit these cellular networks for their own benefit, enhancing viral replication, dissemination in the host or pathogenicity [28].

Previously, we have shown that deletion of SARS-CoV E gene leads to an attenuated virus [18], [19], [21]. In this study, we focused on the contributions of the E protein PBM, a motif that actively participates in protein-protein interactions with host factors [23], to the virulence of SARS-CoV. To this end, different mutant viruses containing altered or deleted E protein PBM sequences were generated using an infectious cDNA clone encoding a SARS-CoV adapted to efficiently grow in mice. Mutant viruses, with or without E protein PBM, grew in Vero E6 and DBT-mACE2 cells with titers similar to those reached by the parental virus. This result indicated that SARS-CoV E protein PBM is not essential for virus replication in cell culture.

Interestingly, recombinant SARS-CoVs lacking E protein PBM were attenuated *in vivo*, causing minimal lung damage, and no mortality in infected mice. In contrast, viruses with functional E protein PBM were highly pathogenic causing 100% mortality and inducing profuse areas of damage in the lung, indicating that E protein PBM is a determinant of pathogenicity. SARS-CoV infection induces an exacerbated immune response that potentiates both epithelial and endothelial damage within the lungs, finally leading to edema accumulation, the ultimate cause of acute lung injury (ALI) and acute respiratory distress syndrome (ARDS) [52]–[54]. Both the enhanced immune response, which leads to cellular infiltration, and edema accumulation leading to pulmonary failure and death, occur when conventional mice are infected with a mouse adapted SARS-CoV [55].

In SARS-CoV-infected patients and animal models, it has been shown that the observed pathology is associated with an exacerbated inflammatory response, linked to elevated levels of pro-inflammatory cytokines [54], [56], [57]. To understand the mechanisms leading to attenuation of viruses lacking E protein PBM, differential host gene expression in mice infected with recombinant viruses with or without an E protein PBM was analyzed using microarray analysis. The expression of genes involved in the innate immune response was significantly reduced in mice infected with SARS-CoV lacking E protein PBM, suggesting an important role of the PBM in the uncontrolled immune response triggered during SARS-CoV infection.

To further understand the molecular basis of the exacerbated immune response induced during SARS-CoV infection in the presence of SARS-CoV E protein PBM, host factors interacting with this motif were identified using a yeast two-hybrid system. One of the most prominent interactions was with the cellular protein syntenin, an important scaffolding protein containing two PDZ domains. PALS1, a protein previously associated to E protein in a similar study [23] was not identified here, probably due to the use of different cDNA libraries. In this work a human lung cDNA library was chosen to further mimic SARS-CoV infection, whereas PALS1 was identified using a human placenta cDNA collection. Coprecipitation analysis revealed that the interaction of syntenin and SARS-CoV E protein was specifically mediated by a functional PBM located in the last 4 amino acid of the E protein, which most likely associates with syntenin PDZ domains, as E protein lacking PBM did not coprecipitate with syntenin. Syntenin may participate in the activation of p38 MAPK [41], a crucial protein involved in the activation of a variety of transcription factors, controlling the expression of genes encoding inflammatory cytokines [42], [43]. Indeed, we have shown an increased expression of inflammatory cytokines *in vivo* during the infection with recombinant SARS-CoVs containing E protein PBM, as compared with viruses lacking this motif, by using microarrays and RT-qPCR assays. Therefore, our results strongly suggest that the interaction of E protein with syntenin induced p38 MAPK activation leading to the inflammatory response observed after SARS-CoV infection. Interestingly, we have shown that in fact, p38 MAPK activation was significantly reduced in mice infected with viruses lacking E protein PBM as compared with mice infected with viruses containing a functional E protein PBM. This reduction in p38 MAPK activation correlated with the acquisition of an attenuated phenotype in SARS-CoV lacking the PBM. In addition, administration of one p38 MAPK inhibitor increased mouse survival after infection with the parental virus. The hypothesis that p38 MAPK is involved in CoV virulence was also postulated when patients with SARS showed augmented p38 MAPK activation [58]. It is clear that multiple host proteins and pathways may be activated through PDZ interactions with SARS-CoV E protein PBM. Interestingly, syntenin and PALS1 interaction with E protein might provide different and even complementary contributions to SARS-CoV pathogenesis, as syntenin has been described in this manuscript that activates p38 MAPK triggering an inflammatory response, whereas PALS1 affects the disruption of the lung epithelium in SARS patients [23].

In agreement with these results, proteins containing PBMs encoded by different viruses such as influenza A virus, tick-borne encephalitis virus (TBEV) and human papillomavirus (HPV) also enhanced virus pathogenesis by interacting with cellular proteins containing PDZ domains, by altering processes such as apoptosis, cell polarity or innate immune responses [28]. Our results are most likely of relevance to other coronaviruses, as the PBM is a highly conserved domain among most coronavirus E proteins, including the highly pathogenic MERS-CoV (Figure S1). Interestingly, although we demonstrated that E protein PBM was not essential for virus production after SARS-CoV infection, not all coronaviruses tolerate amino acid changes in the carboxy-terminus of E protein, as alanine substitutions in MHV E protein carboxy-terminal residues were apparently lethal, since no virus was recovered [59]. Nevertheless, it has not been excluded whether other viral proteins different from E provide alternative PBMs in the cases where the PBM does not seem to be essential.

In summary, we have shown that SARS-CoV E protein contains a functional PBM that contributes to viral pathogenesis and interacts with several cellular proteins, including syntenin. Our studies strongly suggest a causal relationship between E protein-syntenin interaction and p38 MAPK activation, leading to an increase in inflammatory cytokines expression during infection. These data identify syntenin as a potential therapeutic target to reduce the exacerbated immune response induced during SARS-CoV infection. Targeted therapies that temporarily inactivate syntenin or p38 MAPK activation during acute infection may provide a rational approach to improve the prognosis in SARS-CoV patients. In fact, we have shown that inhibition of p38 MAPK increased mice survival after infection with SARS-CoV. Accordingly to our results, *in vivo* inhibition of p38 MAPK diminished influenza virus induced cytokine expression, protecting mice from lethal disease [60]. In the future, a search for the presence of additional PDZ targets located in alternative cellular proteins, and the relevance of E protein PBM present in other coronaviruses will be pursued to better understand the influence of PDZ and PBM motifs in virus pathogenicity and in the immune responses to virus infection.

## Materials and Methods

### Ethics statement

Animal experimental protocols were approved by the Ethical Committee of The Center for Animal Health Research (CISA-INIA) (permit numbers: 2011-009 and 2011-09) in strict accordance with Spanish National Royal Decree (RD 1201/2005) and international EU guidelines 2010/63/UE about protection of animals used for experimentation and other scientific purposes and Spanish National law 32/2007 about animal welfare in their exploitation, transport and sacrifice and also in accordance with the Royal Decree (RD 1201/2005). Infected mice were housed in a ventilated rack (Allentown, NJ).

### Cells

African Green monkey kidney-derived Vero E6 cells were kindly provided by Eric Snijder (Medical Center, University of Leiden, The Netherlands). The delayed brain tumor (DBT) cells expressing mACE2 receptor (DBT-mACE2) were generated in our laboratory [33]. Virus titrations were performed in Vero E6 cells as previously described [18].

### Plasmids

The pcDNA3-E plasmid encoding SARS-CoV E protein was used as previously described [15].The N-terminal HA-tagged syntenin expression plasmid in the backbone of pMT2-HA vector was kindly provided by P.J. Coffer (University Medical Center, Utrecht, The Netherlands) [61].

### Mice

8 week-old specific-pathogen-free BALB/c Ola Hsd mice females were purchased from Harlan Laboratories. BALB/c mice were infected at the age of 16 weeks with 100,000 plaque forming units (pfu).

### Recombinant SARS-CoV growth kinetics

Subconfluent monolayers (90% confluency) of Vero E6 and DBT-mACE2 were infected at an MOI of 0.05 with wt, ΔE, ΔPBM, mutPBM and potPBM. Culture supernatants were collected at different hpi and virus titers were determined as previously described [18].

### Determination of virus titer in infected mouse lungs

Lungs harvested for virus titers were weighed and homogenized using gentleMACS Dissociator (miltenyibiotec). Virus titers were determined by plaque assay on Vero cells as previously described [18].

### Histopathology

Mice were sacrificed at 2 and 4 dpi. Lungs were removed, fixed in zinc formalin and paraffin embedded. Histopathological examinations were performed on hematoxylin-eosin stained sections.

### Western blot analysis

Cell lysates were resolved by sodium dodecyl sulfate-polyacrylamide gel electrophoresis (SDS-PAGE), transferred to a nitrocellulose membrane by wet immunotransfer and processed for Western blotting. The blots were probed with monoclonal antibodies specific for HA tag (dilution 1∶10,000; Sigma), p38 MAPK (dilution 1∶500; Cell Signaling), phospho-p38 MAPK (dilution 1∶500; Cell Signaling), syntenin (dilution 1∶1000; Abcam), phospho-HSP27 (dilution 1∶1000, Cell Signaling) and actin (dilution 1∶10000; Abcam) or polyclonal antibodies against E (dilution 1∶1000), HSP27 (dilution 1∶1000, Cell Signaling) and histone H3 (dilution 1∶5000; Active Motif). A polyclonal antibody recognizing the carboxy-terminal domain of SARS-CoV E protein except the PBM was generated by Biogenes (Germany) using a synthetic peptide corresponding to the 49–64 residues of SARS-CoV E protein (VSLVKPTVYVYSRVKN) as previously described [15]. Bound antibodies were detected with horseradish peroxidase-conjugated goat anti-rabbit or anti-mouse antibodies (dilution 1∶30,000; Cappel) and the Immobilon Western chemiluminescent substrate (Millipore).

### Confocal microscopy

Vero E6 cells were grown to 90% confluency on glass coverslips and infected with the parental virus at an MOI of 0.3. Alternatively, Vero E6 cells were grown to 70% confluency in 1 cm^2^ wells and transfected with 1 µg of DNA using 1 µl of Lipofectamine 2000 (Invitrogen) according to the manufacturer's instructions. At 24 hours post infection (hpi) or post transfection (hpt), cells were fixed as previously described [15]. Primary antibody incubations were performed in PBS containing 10% FBS and 0.2% saponin for 1 h 30 min at room temperature. Immunofluorescence was performed using monoclonal antibodies specific for E (dilution 1∶3000) or N (dilution 1∶500) proteins [15], and polyclonal antibodies specific for syntenin (dilution 1∶200, Abcam). Coverslips were washed four times with PBS between primary and secondary antibody incubations. Alexa 488- or Alexa 546-conjugated antibodies specific for the different species (dilution 1∶500, Invitrogen) were incubated for 45 min at room temperature in PBS containing 10% FBS and 0.2% saponin. Nuclei were stained using DAPI (dilution 1∶200, Sigma). Coverslips were mounted in ProLong Gold anti-fade reagent (Invitrogen) and examined on a Leica SP5 confocal microscope (Leica Microsystems).

### Immunoprecipitation

Vero E6 were grown to 90% confluence and transfected with a N-terminal HA-tagged syntenin expression plasmid. 24 hours later, cells were infected with recombinant viruses at an MOI of 0.3. At 24 hpi, cell extracts were collected as previously described [62]. For immunoprecipitation assays, monoclonal anti-HA agarose conjugate clone HA-7 (Sigma) was used following the manufacturer's instructions. Briefly, 75 µl of the anti-HA agarose conjugate was washed five times with PBS and then incubated with the cell extracts overnight on an orbital shaker at 4°C. The samples were washed four times with PBS and then immune complexes were eluted using 20 µl 2X SDS sample buffer and heating at 95°C for 3 minutes. For reciprocal immunoprecipitation assays Protein A/G Plate IP Kit (Pierce) was used following the manufacturer's instructions as previously described [62] using polyclonal anti-E antibody. Analysis of precipitate complexes was carried out by SDS-PAGE and Western blotting.

### Cytokine expression analysis from lung samples using RT-qPCR

Lung sections from infected animals were collected at 2 dpi and homogenized using gentleMACS Dissociator (Miltenyibiotec). Then, total RNA was extracted using the RNeasy purification kit (Qiagen). Reactions were performed at 37°C for 2 h using a High Capacity cDNA transcription kit (Applied Biosystems) using 100 ng of total RNA and random hexamer oligonucleotides. Cellular gene expression was analyzed using TaqMan gene expression assays (Applied Biosystems) specific for mouse genes (Table 1). Data representing the average of three independent experiments were acquired and analyzed as previously described [32]. All experiments and data analysis were MIQE compliant [63].

**Table 1:** Taqman assays used to analyze the expression of cellular genes by quantitative RT-PCR.

Gene name	Taqman assay*	Description
*CXCL10/IP-10*	Mm00445235-m1	Interferon inducible protein 10
*CCL2/MCP-1*	Mm00441242-m1	Monocyte chemotactic protein 1
*IL-6*	Mm00446190-m1	Interleukin 6
*18S*	Mm03928990-g1	18S rRNA
*SDCBP (syntenin)*	Hs01045460_g1	Syndecan binding protein (syntenin)

*Mm, means *Mus musculus*. Hs, means *Homo sapiens*.

### Yeast two-hybrid screening

Bait cloning and yeast two-hybrid screening with the carboxy-terminal (amino acids 36–76) domain of SARS-CoV E protein (E_CT_) as bait were performed by Hybrigenics (France). E_CT_ domain was cloned into the pB27 vector, enabling its fusion with the LexA binding domain. The bait construct was transformed into the L40ΔGAL4 yeast strain [64] and then mated with the Y187 yeast strain transformed by a random-primed human lung cDNA library containing 10 million independent fragments. In the screening, 80.3 million interactions were analyzed. After selection on medium lacking leucine, tryptophan, and histidine, 268 positive clones were picked. The corresponding prey fragments were subjected to PCR and sequencing. Sequences were then filtered, divided into contigs, and compared to the latest release of the GenBank database by using BLASTn (NCBI). A predicted biological score (PBS) was attributed to assess the reliability of the interaction, as described earlier [38].

### p38 MAPK activation in lungs of infected mice

Lungs were removed from infected mice at 2 dpi and homogenized. Nuclear and cytoplasmic extracts from homogenized lungs were obtained using a nuclear extract kit (Active Motif, Carlsbad, CA). Levels of total and phosphorylated p38 MAPK were analyzed by Western blot using specific antibodies and the cytoplasmic extracts. Total and activated p38 MAPK amounts were quantified by densitometric analysis using Quantity One, version 4.5.1, software (Bio-Rad). In each case, the levels of phosphorylated p38 MAPK were normalized to the levels of total p38 MAPK. Three different experiments and appropriate gel exposures were used in all cases with similar results. In addition, different exposures of the same experiment were analyzed to assure that data obtained were within linear range.

### Microarray analysis

At 2 days post infection, lungs from infected mice were collected and homogenized using the gentleMACS Dissociator (Miltenyibiotec). Then, total RNA was extracted using the RNeasy purification kit (Qiagen) according to the manufacturer's instructions. Three biological replicates were independently hybridized for each transcriptomic comparison. Total RNA (200 ng) was amplified using One Color Low Input Quick Amp Labeling Kit (Agilent Technologies) and purified with RNeasy Mini Kit (Qiagen). Preparation of probes and hybridization was performed as described in One-Color Microarray Based Gene Expression Analysis Manual Ver. 6.5, Agilent Technologies. Briefly, for each hybridization 600 ng of Cy3 probes were mixed and added to 5 ul of 10x Blocking Agent, 1 ul of 25x Fragmentation Buffer and Nuclease free water in a 25 µl reaction, incubated at 60°C for 30 minutes to fragment RNA and stopped with 25 ul of 2x Hybridization Buffer. The samples were placed on ice and quickly loaded onto arrays, hybridized at 65°C for 17 hours in a Hybridization oven rotator and then washed in GE wash buffer 1 at room temperature (1 minute) and in GE Wash Buffer 2 at 37°C (1 minute). Arrays were dried by centrifugation at 2000 rpm for 2 minutes. Slides were Sure Print G3 Agilent 8×60K Mouse (G4852A-028005)

Images were captured with an Agilent Microarray Scanner and spots quantified using Feature Extraction Software (Agilent Technologies). Background correction and normalization of expression data were performed using LIMMA [65].

### Microarray data analysis

Linear model methods were used for determining differentially expressed genes. Each probe was tested for changes in expression over replicates by using an empirical Bayes moderated t-statistic [66]. To control the false discovery rate (FDR), defined as the expected proportion of false positives among the significant tests, *p-values* were corrected by using the method of Benjamini and Hochberg [66], [67]. The expected false discovery rate was controlled to be less than 5% (FDR<0.05). Genes were considered differentially expressed when the FDR were <0.01. In addition, only genes with a fold change of >2 or of <−2 were considered for further analysis.

### Viruses

The mouse-adapted (MA15) [31], parental virus (wt) and a virus lacking E gene (ΔE) were rescued from infectious cDNA clones generated in our laboratory [21].

The pBAC-SARS-CoV-E-PBM mutant plasmids encoding recombinant SARS-CoVs expressing E genes with deleted or mutated PBMs were constructed from a previously generated infectious cDNA clone (plasmid pBAC-SARS-CoV-ΔE-MA15) [21]. Deletion of the 9 most carboxy-terminal amino acids (LNS---------) or mutations (LNSSAGAPALAV) in SARS-CoV-E-ΔPBM and SARS-CoV-E-potPBM, respectively (Figure 1), were introduced by overlap extension PCR using the pBAC-SARS-CoV-ΔE-MA15 as a template and specific primers (Table 2). A fragment representing the nucleotides containing the mutations (LNSSEGVPGGGG) to generate SARS-CoV-E-mutPBM was chemically synthesized (BioBasic Inc). The final PCR products and synthesis fragments were digested with enzymes *BamHI* and *MfeI* and cloned into the intermediate plasmid psl1190+BamHI/SacII-SARS-CoV to generate the plasmids psl1190-E-ΔPBM, psl1190-E-mutPBM and psl1190-E-potPBM. The plasmid psl1190+BamHI/SacII SARS-CoV contains a fragment corresponding to nucleotides 26045 to 30091 of the SARS-CoV infectious cDNA clone [68] engineered into plasmid psl1190 (Pharmacia). These constructs were cloned in the infectious pBAC-SARS-CoV-ΔE-MA15 with the enzymes *BamHI* and *SacII*. All constructs were generated carrying a duplication of the final last nucleotides (208–231) of E gene after the stop codon of the mutated E proteins in order to avoid altering the transcription regulatory sequence (TRS) of membrane (M) gene, which overlaps with the end of E gene [69]. All viruses were rescued from infectious cDNA clones as previously described [68].

**Table 2:** Primers used for the generation of recombinant SARS-CoV-E-PBM mutants.

Virus	Primer	Sequence
**ΔPBM**	SARS-25871-VS	CGTTGTACATGGCTATTTCACCG
	SARS-ΔPBM-RS	TTAGACCAGAAGATCAGGAACTCCTTAAGAGTTCAGATTTTTAACACGC
	SARS-26325-VS	GGAGTTCCTGATCTTCTGGTCTAA
	SARS-28136-RS	GGGCACTACGTTGGTTTGATTGGGG
**potPBM**	SARS-25871-VS	CGTTGTACATGGCTATTTCACCG
	SARS-potPBM-RS	TTAGACCAGAAGATCAGGAACTCCTTAGACTGCAAGAGCAGGAGCTCCTGCAGAAGAGTTCAGATTTTTAACACGCG
	SARS-26325-VS	GGAGTTCCTGATCTTCTGGTCTAA
	SARS-28136-RS	GGGCACTACGTTGGTTTGATTGGGG

### siRNA transfection

Vero E6 cells were transfected following a reverse transfection protocol. Briefly, for each well of a 24-well plate, 5×10^4^ cells were incubated in suspension with 25 nM of Silencer Select siRNAs (Ambion) targeting syntenin (siRNA 1: s12641 and siRNA 2: s224582) and 2 µl of siPORT amine (Ambion) diluted in 50 µl of Opti-MEM I reduced serum medium (GibcoBRL-Invitrogen), following the manufacture's instructions. As a negative control, an irrelevant validated siRNA (Ambion, reference 4390843) was transfected. Cells were plated onto each well using DMEM with 5% heat-inactivated FBS, incubated at 37°C for 24 h. The cells were retransfected with 25 nM siRNAs at 24 h after the first transfection, and infected with the parental virus at 48 h after second transfection. At 24 hpi, total RNA, proteins and cells supernatants were collected for further analysis.

### Analysis of syntenin gene expression

Syntenin gene expression was quantified by qRT-PCR. Total RNA was prepared with an RNeasy kit (Qiagen), according to the manufacturer's instructions. Reactions were performed at 37°C for 2 h using a High Capacity cDNA transcription kit (Applied Biosystems) using 100 ng of total RNA and random hexamer oligonucleotides. Syntenin gene expression was analyzed using TaqMan gene expression assays (Applied Biosystems) specific for human gene (Table 1). Data representing the average of three independent experiments were acquired and analyzed as previously described [32]. All experiments and data analysis were MIQE compliant [63].

### p38 MAPK inhibitor treatment of SARS-CoV-infected BALB/c mice

16-week-old BALB/c mice were infected intranasally with 100,000 pfu of wt virus. At 4 hpi and every 12 h thereafter, from days 1 to 8, mice were treated intraperitoneally with SB203580 (Millipore) at 6 mg/kg of body weight/day with vehicle (PBS containing 2% dimethyl sulfoxide [DMSO]). Survival was analyzed in three independent experiments with 5 mice per group. To analyze p38 MAPK inhibition, lungs were removed and homogenized from mice at 2 dpi. Levels of total and phosphorylated HSP27 were analyzed by Western blot using specific antibodies and the lung extracts. Total and activated HSP27 amounts were quantified by densitometric analysis using Quantity One, version 4.5.1, software (Bio-Rad). In each case, the levels of phosphorylated HSP27 were normalized to the levels of total HSP27. Three different experiments and appropriate gel exposures were used in all cases with similar results. In addition, different exposures of the same experiment were analyzed to assure that data obtained were within linear range.

### Subcellular fractionation

Vero E6 were washed twice with PBS, scraped off and pelleted by low-speed centrifugation at 2000 rpm for 2 minutes in a bench-top centrifuge. The supernatant was removed and cell pellets were lysed by repetitive pipetting with a micropipette in ice-cold lysis buffer containing 150 mM NaCl, 3 mM MgCl_2_, 20 mM Tris/HCl (pH 7.5), 2 mM DTT and 0.5% NP-40. The lysate was incubated during 5 minutes in ice and centrifuged in a bench-top centrifuge at 3000 rpm for 2 minutes. Supernatant and pellet were saved as the cytosolic and nuclear fraction, respectively.

### Scoring of lung pathology

Hematoxylin and eosin-stained lung sections were assessed using the scoring system described in the figure legends according to previously described procedures [34]. Three animals for each time point were analyzed.

### Accession numbers

The UniProt (http://www.uniprot.org/) accession numbers for genes and proteins discussed in this paper are: SARS-CoV E protein, P59637; human p38 MAPK, Q16539; mouse p38 MAPK, P47811; syntenin, O00560; human ACE2, Q9BYF1; mouse ACE2, Q8R0I0; PALS1, Q8N3R9; SAA2, P05367; CCL3, P10855; CXCL1, P12850; CXCL5, P50228; CALCA, P70160; SAA1, P05366; CXCL10, P17515; CCL2, P10148; IL1B, P10749; ORM1, Q60590; IL6, P08505; CCL4, P14097; CXCL9, P18340; 18S, O35130; human actin, P60709; mouse actin, P60710; histone H3, P84243; SARS-CoV N protein, P59595; HSP27, P14602.

## Supporting Information

Figure S1 **Coronavirus E protein sequences representing potential PDZ-binding motifs.** Top, representation of CoV E protein sequence and its corresponding domains. Below sequences corresponding to the end of several E proteins from representative genus *α*, *β* and *γ* CoVs are shown in boxes. Red boxes represent the presence of a potential PDZ-binding motif.
(TIF)
