# Supplementary figures and images for: The PDZ-Binding Motif of Severe Acute Respiratory Syndrome Coronavirus Envelope Protein Is a Determinant of Viral Pathogenesis
Source: PLoS Pathog. 2014 Aug 14;10(8):e1004320. doi: 10.1371/journal.ppat.1004320 (PMC4133396; doi:10.1371/journal.ppat.1004320)

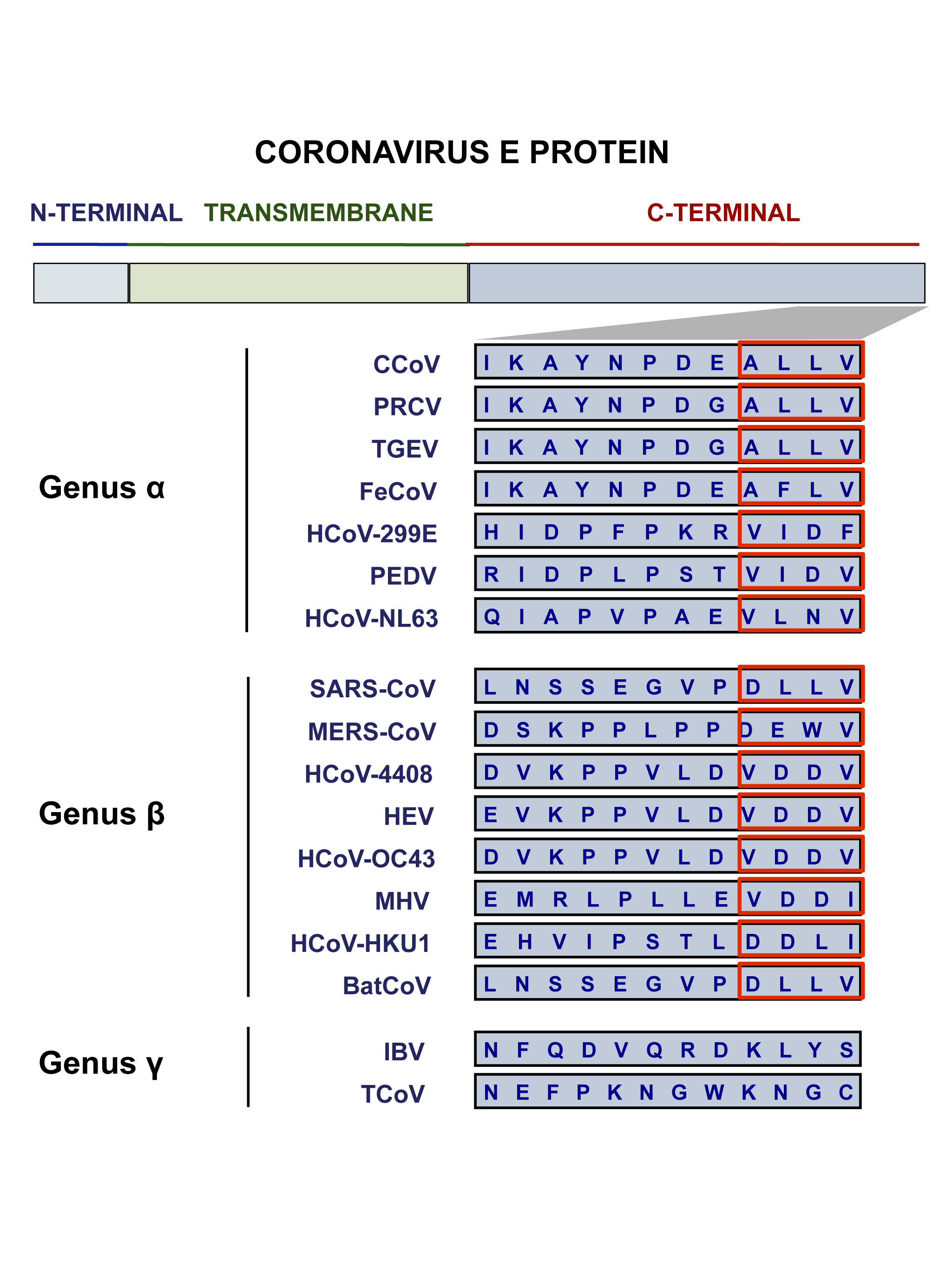

Supplement: Figure S1 — Coronavirus E protein sequences representing potential PDZ-binding motifs. Top, representation of CoV E protein sequence and its corresponding domains. Below sequences corresponding to the end of several E proteins from representative genus α, β and γ CoVs are shown in boxes. Red boxes represent the presence of a potential PDZ-binding motif. (TIF) [file ppat.1004320.s001.tif]
